# Assessment of Laser Parameters to Improve Lid Tension—A Proof of Concept towards Lasercanthoplasty

**DOI:** 10.3390/ijms24054757

**Published:** 2023-03-01

**Authors:** Christoph Holtmann, Joana Witt, Alexandra Schilcher, Amar Avdakovic, Alessa Hutfilz, Dirk Theisen-Kunde, Katharina Wiebe-Ben Zakour, Erich Knop, Gerd Geerling

**Affiliations:** 1 Department of Ophthalmology, University Hospital, Heinrich-Heine-University Duesseldorf, Moorenstr. 5, 40225 Duesseldorf, Germany; 2 Medical Laser Center Luebeck, Peter-Monnik-Weg 4, 23562 Luebeck, Germany; 3 Department of Anatomy, Health and Medical University (HMU) Potsdam, Olympischer Weg 1, 14471 Potsdam, Germany

**Keywords:** dry eye, ocular surface, meibomian gland dysfunction, lid tension, laser treatment, basic research

## Abstract

Background: Preliminary clinical work indicates that increasing eyelid tension improves the function of the meibomian glands. The aim of this study was to optimize laser parameters for a minimally invasive laser treatment to increase eyelid tension by coagulation of the lateral tarsal plate and canthus. Methods: Experiments were performed on a total of 24 porcine lower lids post mortem, with six lids in each group. Three groups were irradiated with an infrared B radiation laser. Laser-induced lower eyelid shortening was measured and the increase in eyelid tension was assessed with a force sensor. A histology was performed to evaluate coagulation size and laser-induced tissue damage. Results: In all three groups, a significant shortening of the eyelids after irradiation was noticed (*p* < 0.0001). The strongest effect was seen with 1940 nm/1 W/5 s, showing −15.1 ± 3.7% and −2.5 ± 0.6 mm lid shortening. The largest significant increase in eyelid tension was seen after placing the third coagulation. Conclusion: Laser coagulation leads to lower eyelid shortening and an increase in lower eyelid tension. The strongest effect with the least tissue damage was shown for laser parameters of 1470 nm/2.5 W/2 s. In vivo studies of this effect have to confirm the efficacy of this concept prior to clinical application.

## 1. Introduction

Meibomian glands produce a lipid secretion that is liquid at body temperature and thus forms an oil that is delivered onto the posterior lid margin, and from there onto the surface of the tear film [1]. They have several functions for the stability and rheology of the tear film. Presently available findings indicate that one of the most important functions of the meibomian oil is to retard the evaporation of the main aqueous phase of the tears. Several recent independent studies have shown that a primary oil deficiency due to meibomian gland dysfunction (MGD) is not only the major factor of evaporative dry eye (EDE), but also constitutes the most frequent cause of dry eye disease (DED) in general [2,3]. The tarsus and canthal tendons are responsible for the main passive tensile strength of the eyelids and their stable shape. The stability of the tarsal plate is crucial for the equal transmission of the mechanical force of the orbicularis muscle during a blink onto the meibomian glands, and thus for expression of the oil out of the gland orifices onto the lid margin. 

So far, very few publications mention the meibomian glands, tear film parameters, or symptoms of DED with reference to eyelid tension. Preliminary clinical work of our group in patients with laxity of the eyelids has indicated that increasing eyelid tension does indeed improve the function of the meibomian glands [4]. When laxity of the eyelids was surgically corrected with a lateral tarsal strip procedure, which shortens the lower lid and re-fixes it at the lateral orbital wall, signs and symptoms (using the validated OSDI questionnaire) of MGD improved three months postoperatively. 

Aside from addressing lid laxity by means of a simple open surgery, the eyelid could also be shortened and lid tension increased by using a laser application for controlled collagen coagulation e.g., of the lateral canthal area (Lasercanthoplasty (LCP)). Due to its minimally invasive nature, this concept of transepithelial laser induced tissue shrinkage could offer the advantage of being minimally invasive and potentially easily repeatable. The aim of this study was to assess and optimize laser treatment parameters to increase the eyelid tension by coagulation of the lateral tarsal plate and the canthal tendon. Dose finding experiments for increasing eyelid tensile strength by means of an infrared B radiation laser were performed on ex vivo healthy porcine lower lids. To evaluate the impact of a controlled coagulation of the extracellular matrix of the eyelid and to determine possible adverse effects on the conjunctival epithelium and stroma, a tension analysis during and after the procedure, as well as a histology, was performed.

## 2. Results

### 2.1. Eyelid Shortening and Changes in Eyelid Tension after Laser Coagulation

**Figure 1:**
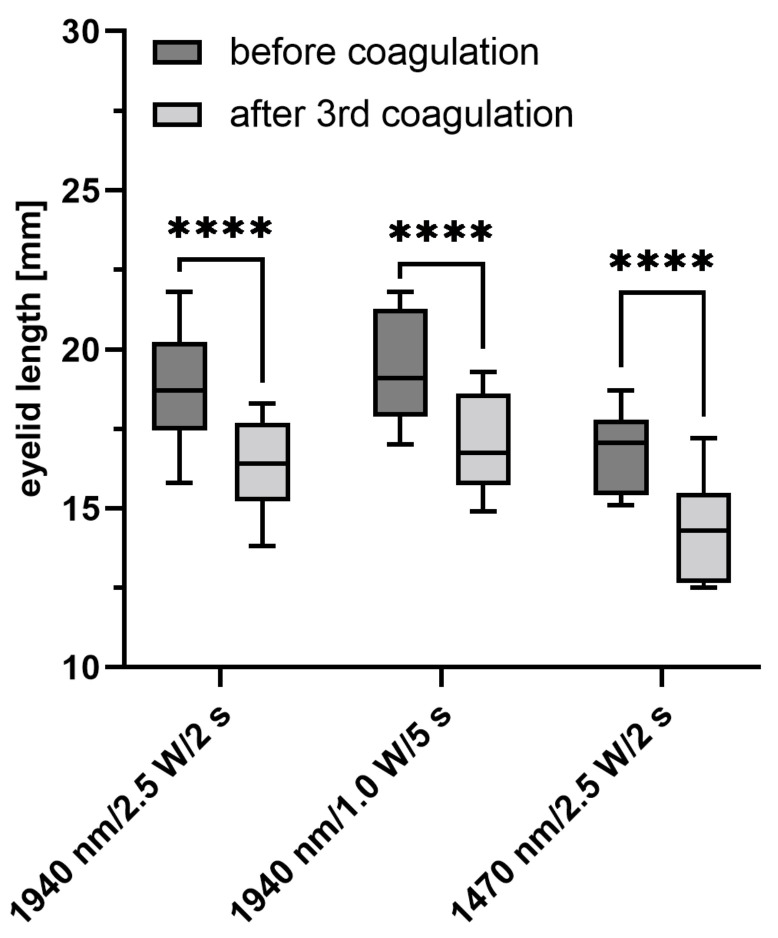
Changes in eyelid length before and after irradiation using three different laser settings. **** *p* < 0.0001.

In all three groups of laser parameter combinations, a significant shortening of the eyelids after irradiation compared to the control group was noticed (*p* < 0.0001) (Figure 1). For the groups with 1940 nm wavelength, longitudinal shortening was almost identical (Group I: −12.9 ± 2.7% −2.4 ± 0.7 mm; Group II: −12.3 ± 0.6% ≙ −2.4 ±0.3 mm; *p* ≥ 0.88). The strongest shortening was seen in the 1470-nm-group III, with −15.1 ± 3.7% ≙ −2.5 ± 0.6 mm.

**Figure 2:**
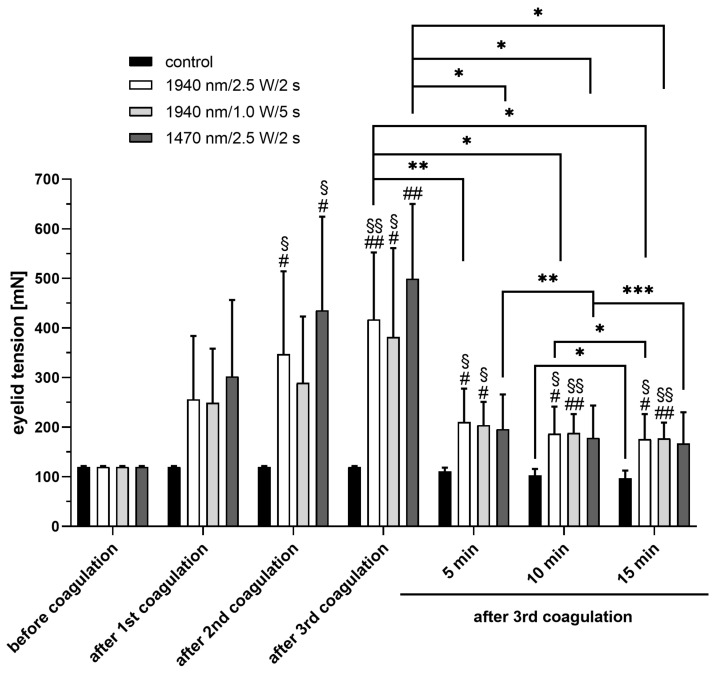
Changes in eyelid tension before and after irradiation using three different laser settings. # *p* < 0.05, ## *p* < 0.01 compared to before coagulation. § *p* < 0.05, §§ *p* < 0.01 compared with control group. * *p* < 0.05, ** *p* < 0.01, *** *p* < 0.001.

The eyelid tension before and after irradiation was compared, as well as the tension after irradiation to a control group, in which the eyelids were not irradiated but clamped in the material testing machine during the experiment (Figure 2). No significant increase in eyelid tension was detected after placement of the first coagulation compared with the untreated group before irradiation (Group I *p* = 0.28; group II: *p* = 0.22; group III: *p* = 0.21). The first significant increase was observed after placing the second coagulation in group I (*p* = 0.05) and group III (*p* = 0.03). The largest significant difference to the eyelid tension before irradiation was observed after the placement of the third coagulation. (Group I: *p* = 0.01; group II: *p* = 0.04. group III: 499.3 ± 150.5 mN; *p* = 0.004). Five minutes after laser application, there was a sharp decrease in tissue tension in all three groups compared to the tension immediately after the third coagulation. However, this was only significant in group I and III (*p* = 0.01; *p* = 0.014, respectively). Lid tension decreased slightly more with time; however, 15 min after application, the tension in group I and II was still higher than before the irradiation (*p* = 0.02; *p* = 0.002, respectively). There were no significant differences between the three different treatment groups immediately after irradiation or 5, 10 or 15 min later. A slight decrease of eyelid tension in non-lasered eyelids in the control group was observed. There was a significant decrease in eyelid tension from minute 10 to 15 after setting the third coagulation (*p* = 0.046). However, the difference compared with the tension before irradiation was not significant.

### 2.2. Histological Changes of Eyelids after Laser Coagulation

**Figure 3:**
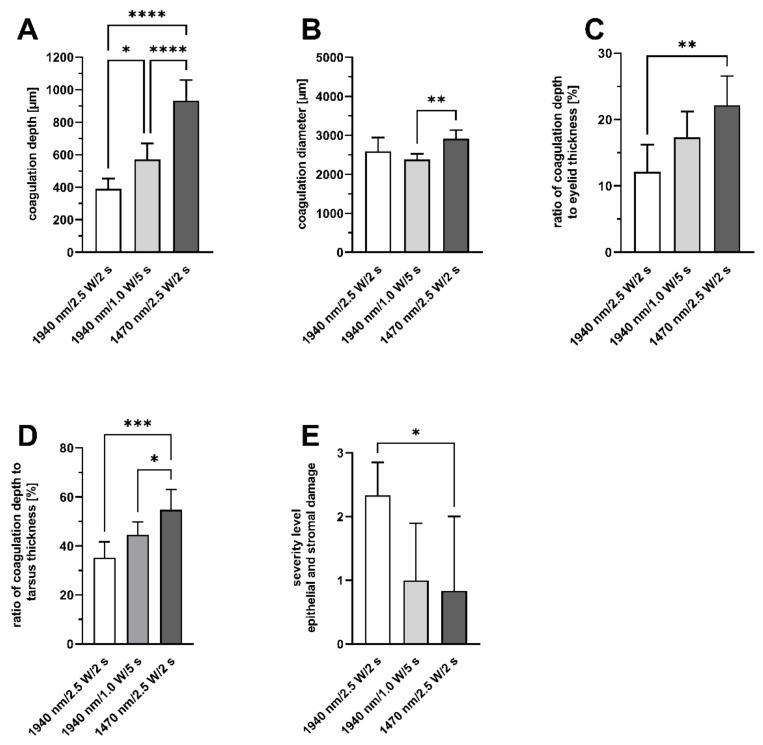
Histological results of laser coagulations of porcine eyelids in a histological cross section. (**A**): Depth of coagulation. (**B**): Diameter of coagulation (measured at the epithelium). (**C**): Percentage of coagulation depth to lid thickness. (**D**): Percentage of coagulation depth to tarsus thickness. (**E**): Severity of epithelial and stromal damage. * *p* < 0.04 ** *p* < 0.007 *** *p* < 0.002; **** *p* < 0.0001.

Coagulation was significantly deeper in group III with 1071.8 ± 70.8 µm than in group I with 389.9 ± 63.2 µm (*p* < 0.0001) or in group II with 571.2 ± 98.2 µm (*p* < 0.0001) (Figure 3A). The difference of coagulation depth of group I and II was also significant (*p* = 0.017).

Evaluation of the coagulation diameter measured at the epithelium (Figure 3B) showed significant differences between group II (2380.1 ± 146.9 µm) and III (2910.8 ± 220.6 µm) (*p* = 0.007). The coagulation diameter in group I was 2592.1 ± 352.8 µm, and was not significantly different to group II or III (*p* ≥ 0.12). 

The average thickness of all eyelids measured in HE-stained cross-sections was 3.71 ± 0.49 mm. The ratio of the coagulation depth to the total eyelid thickness was 22.17 ± 4.39% in group III, the highest among all groups, and significantly higher than in group I, which had the lowest value of 12.1 ± 4.13% (*p* = 0.002). (Figure 3C). In group II, the coagulation-depth-to-lid-thickness ratio was 17.3 ± 3.9%. There was no statistically significant difference compared to group I or III (*p* ≥ 0.11). 

Evaluation of the ratio of coagulation depth to the tarsus thickness showed similar results: group I: 35.3 ± 6.4%; group II: 44.6 ± 5.3%; group III: 59.1 ± 8.3% (Figure 3E). There was a significant difference between group I and III (*p* = 0.0004) and between group II and III (*p* = 0.05) 

**Figure 4:**
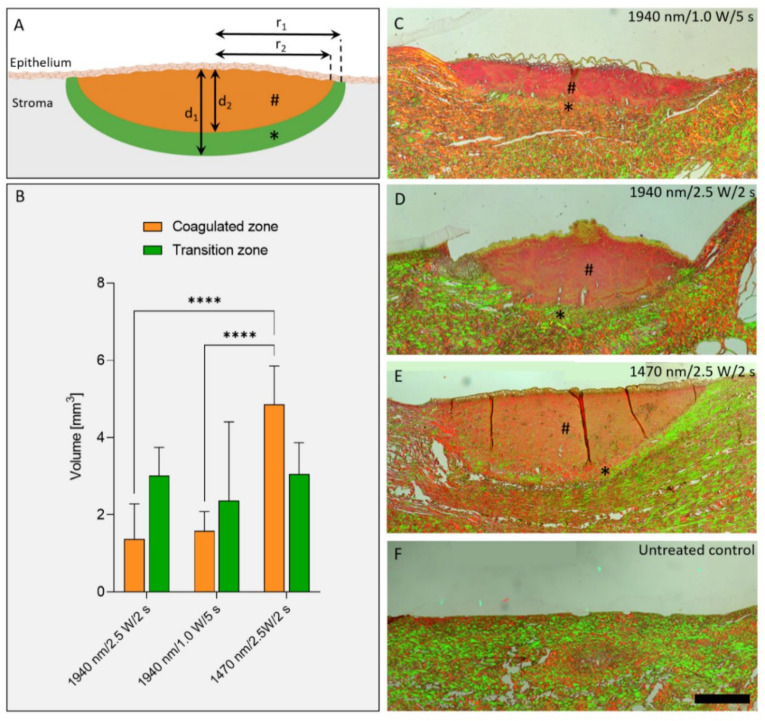
Evaluation of the thermally altered zones of laser damage of porcine eyelids. The coagulated zone (#) and the transition zone (*) was determined (**A**), and the volume of both zones was calculated (**B**) using the depth and the radius measured in Picro-Sirius-Red-stained cross-sections (**C**–**F**). In the transition zone, stronger birefringence was observed compared to the uncoagulated normal tissue. d_1_ = depth coagulated zone plus transition zone; d_2_ = depth coagulated zone; r_1_ = radius coagulated zone plus transition zone; r_2_ = radius coagulated zone. **** *p* < 0.0001. Scale bar: 500 µm.

A coagulated zone and a transition zone were distinguished in Picro-Sirius Red stained sections observed under polarized light (Figure 4). The coagulated zone (marked with #) was characterized by a complete loss of birefringence and the random rearrangement of parallel collagen fibrils by laser application. This disordered collagen was darker. The transition zone (marked with *) showed stronger birefringence compared to the normal, non-coagulated tissue. The calculated volume of coagulated tissue of the group with the wavelength of 1470 nm was significantly larger (*p* < 0.0001) than in the other two groups (group I: 1.37 ± 0.91 mm^3^; group II: 1.58 ± 0.50 mm^3^; group III: 4.86 ± 0.99 mm^3^). However, the transition zone in the 1470 nm wavelength group was comparable to the 1940-nm-groups (group I: 3.01 ± 0.73 mm^3^; group II: 2.37 ± 2.03 mm^3^; group III: 3.05 ± 0.82 mm^3^; all *p* ≥ 0.65).

## 3. Discussion

In the study presented here, we investigated different laser parameters as a proof of concept for a minimally invasive procedure (laser -canthoplasty) to increase eyelid tension by collagen coagulation.

Scattering and absorption in tissue are wavelength dependent. One of the main absorbers at 1470 and 1940 nm in a living tissue is water. The wavelengths differ in their absorption coefficient (27.6 ${\mathrm{cm}}^{-1}$ and 128 ${\mathrm{cm}}^{-1},\;\mathrm{respectively}$) in water [5]. 

The Intensity *I_x_* in a depth (*x*) can be calculated by

$$I_{x}=I_{0}*e^{-\alpha *x}$$

with *I*_0_ as incident intensity and *α* as absorption coefficient. A higher absorption coefficient yields to a higher energy absorption within the distance *x*. Assuming that most of the energy is converted into heat means that the higher the absorption coefficient, the more heat that is generated in the same tissue volume within a certain depth. Therefore, the wanted tissue effect (small or high temperature gradient) depends on the absorption coefficient and therefore to the wavelength. The two wavelengths were chosen to be compared based on the fact that it was not clear which coagulation zone was best for the desired effect. 

The threshold for tissue damage further depends on the laser power (temperature) and the irradiation time. This threshold can be reached by a high laser power in a short time, or with a lower laser power and a longer irradiation time. In this work, both effects were investigated. The laser power was 1 and 2.5 W for the wavelength of 1940 nm. The irradiation time varied between 2 and 5 s. For the group with a wavelength of 1470 nm, the irradiation time of 2 s was considered with a laser power of 2.5 W. The two wavelengths were chosen because of their high absorption coefficient in water. According to the relationship of laser power and irradiation time, the irradiation energy was 5 J for all groups. In preliminary investigations at the Medical Laser Center Luebeck, this irradiation energy was determined for the continuous application mode. Higher irradiation energies led to the carbonization of the lower eyelids. Longer irradiation times (10 s) at low power (0.5 W) showed no measurable changes in eyelid tension. Previous works from members of our group showed that in the human cornea, tissue damage depended on laser power, the absorption coefficient, and the corneal thickness. With increasing laser power, the penetration depth of the coagulation increased, while at the same time endothelial cell damage increased as well [6,7,8]. As there are different phases of collagen in transition as well as a complex time-temperature-behavior during laser exposure, further optimization of laser parameters have yet to be obtained [8,9,10]. In the human cornea, the course of contractive forces during and after heating is a complicated function of a spatial time/temperature profile. In laser thermokeratoplasty, lesions produced with two irradiation times showed different stages of denaturation and therefore induced refractive change [11]. With a well-adapted focusing device, in diode laser thermokeratoplasty the refractive change increased almost logarithmically with the irradiation time up to 15 s [12]. In human skin, Paul Ruff was able to show that a plasma and radiofrequency system was effective and safe for the treatment of submental laxity [13].

The controlled coagulation of the extracellular matrix of the porcine eyelids showed variable longitudinal shortening as well as laser-induced damage to the tissue. After irradiation, the most extensive longitudinal shortening as well as the increase in lower eyelid tension after the third coagulation was shown for group 3 (1470 nm/2.5 W/2 s). This group also showed the highest percentage of coagulation within the total diameter of the tarsus, with the least epithelial changes and deeper tissue destruction. In an ex vivo sheep model, the effect of collagen coagulation with UV-A radiation (365 nm) on lower eyelid tension was previously published by Smith et al. in 2018 [14]. They were able to achieve increased tensile strength of the tarsal plate without destruction of the meibomian glands and collagen structures. The results showed that irradiation induced both the stiffening and the strength increase of the tarsal plate. This effect was evident at low (3 or 6 mW/cm^2^) as well as higher irradiances (30 or 45 mW/cm^2^), with the effect increasing at higher irradiances. In another investigation, the same group concluded that the irradiation of tarsal collagen leading to tissue stiffening shall be carried out at fluence levels between 10 and 15 J/cm, while the exposure time can vary [15]. Changes after crosslinking in human specimens (after wedge excision) seem to increase grossly, but changes in tensile properties were not found to be statistically significant [16]. Nonetheless, a variety of negative effects on the human organism have been described for UV radiation due to its high energy, such as premature skin aging, skin cancer, and damage to the eyes (cataracts).

Five minutes after coagulation, lid tension decreased again in all of our groups, with no significant changes between these groups. A slight decrease in tension over time was also observed in the untreated control group, which suggests a progressive stretching of the clamped tissue. Furthermore, in the human cornea, tissue regression was a setback in the long term effects of the procedure [9]. In the skin of the neck, a minimally invasive soft tissue tightening technique using subcutaneous and subdermal radiofrequency-assisted lipocontouring (RFAL) showed as much as a 25% area contraction at 6 months and 35–40% achieved at 1 year [17].

The absorption coefficient for water at 1940 nm with 128 cm^−1^ is about a factor of five higher than at 1470 nm with 27.6 cm^−1^ [5]. Therefore, light with a wavelength of 1470 nm penetrates deeper into the tissue, which should result in a larger coagulation zone than light with a wavelength of 1970 nm. This study confirmed these basic principles of laser-tissues interaction, as well as the assumption that such laser induced coagulations can be used to increase horizontal lid tension.

In order to evaluate the depth of the coagulation defect according to a power and irradiation time of 1470 nm diode laser on the perianal tissue model, Danys et al. found that the longest tissue injury was caused by the longest laser exposure time, with no significant difference between laser power used [18]. As we reduced the power by 2.5-fold and simultaneously increased the duration by the same amount, the coagulation depth of group II and III behaved very similarly. Giglio and Fried used a 1470 nm laser for the sealing and dissection of blood vessels, and they evaluated optical, thermal, and tissue damage [19]. They found that, the yielding thermal spread increased with higher power (up to 200 W), as was expected. With the power chosen in our experiments (2.5 W), the overall tissue damage was very limited. Troung et al. investigated the effect of radial and cylindrical light distributions on the response of vascular tissue during 1470-nm irradiation in ex vivo models [20]. Low irradiance (5.3 W/cm^2^) and wide light distribution, in addition to cylindrically diffusing irradiation, yielded uniform thermal coagulation in leporine veins. Even though the power used in our experiments was further reduced, this low irradiance showed better controlled coagulation with limited tissue damage. Tran and colleagues also tested a 1470-nm laser light in various irradiation conditions to evaluate the thermal gradient and temperature evolution in the trachea ex vivo [21]. They were able to show a tissue shrinkage of up to 16.5%. While the overall power and duration of laser irradiation was lower in our experiments, we also observed detached conjunctival epithelium, especially in the central area of laser coagulation.

Overall, a minimal invasive laserkanthoplasty procedure would have advantages over a conventional lid tightening surgical procedure. If the size and handling of the device allow it, this procedure could be performed at the bedside under non-sterile conditions, and possibly with only a topical anesthetic. The procedure could be repeated if adequate lid tension was not achieved after a single session. Comparable to other laser procedures, laser parameters and the number of laser coagulations can be varied based on the severity of the lid laxity.

Our work is limited by several factors. First, the eyelids were marked with a blue skin marking pen before the laser spots were placed. Although the wavelength of the laser was not in the blue wavelength range, non-tissue-dependent energy absorption of the blue dots cannot be completely excluded. The experiments to evaluate the influence of different laser parameters (wavelength, power, irradiation time) on the porcine lids ex vivo were restricted to three treatment groups, and no further treatment modalities were examined. Moreover, the number of tested specimens was limited in each group, and eyelid shortening and tension in particular were measured for 15 min post treatment only.

## 4. Material and Methods

Experiments were performed on porcine lower eyelids provided immediately post mortem by a local abattoir. The tissue samples were stored on ice until use.

### 4.1. Laser Application on Porcine Eyelids

**Figure 5:**
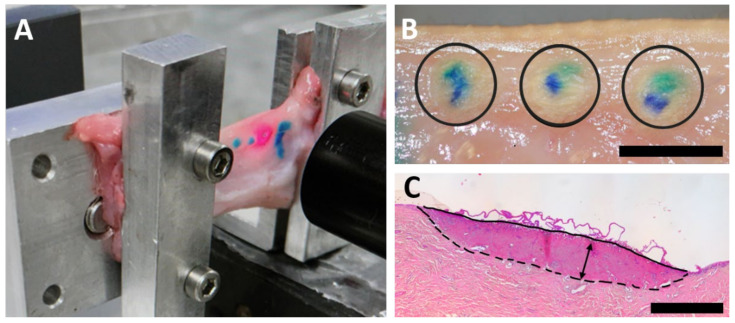
(**A**) Illustration of the porcine lower eyelid tissue fastened between clamps to measure the tension during the experiment (**B**): Laser coagulations (black circles) after application. (**C**): Coagulation size was determined in HE staining. Arrow: Depth of coagulation, black line: coagulation diameter, epithelial side; dashed line: coagulation diameter, stromal side. Scale bars represent 5 mm (**B**) and 500 µm (**C**).

For laser application, the eyelids were fixed between two metal clamps on each side, connected to a force sensor (S 524060, LD Didactic GmbH, Hürth, Germany) with a base tension of 120 mN. (Figure 5A). The force sensor continuously measured the tension and its changes in the eyelid during and after laser treatment. To ensure physiological tissue hydration, the conjunctiva of the porcine lid was dripped with 2 mL of 0.9% sodium chloride throughout the experiment.

Before laser irradiation, the application laser sites were marked with a blue skin-marking pen (Figure 5A). Three laser coagulations were subsequently placed on the conjunctiva overlying the tarsal plate along the lateral eyelid margin, with 15 mm vertical distance to the lid margin and 5 mm between coagulation sites (Figure 5B).

The laser system (MultiPulse Tm + 1470, Asclepion Laser Technologie GmbH, Jena, Germany) was provided by the Medical Laser Center Luebeck. The laser source (MultiPulse Tm + 1470, Asclepion Laser Technologie GmbH, Jena, Germany) emits infrared B radiation with wavelengths of 1470 and 1940 nm. A collimator (laboratory-build) was used to provide an approximately parallel beam of 3 mm in diameter. Irradiation was applied at a perpendicular distance of 2 cm to the conjunctival side of the eyelids. A time-controlled laser shutter was used to determine the irradiation time. All treatment groups (n = 6) were irradiated in a continuous wave mode. Based on the properties of the laser, different laser parameter combinations were chosen for irradiation on the eyelids (Table 1).

The applied irradiation energy for each coagulation spot remained constant at 5 J, since higher energies caused carbonization of the tissue in preliminary tests, which are not discussed in this manuscript. The experiments were performed on 24 porcine lower eyelids, with each treatment group including six individual eyelids (n = 6). The control group (n = 6) was not irradiated.

### 4.2. Measurement of Eyelid Tension

For the quantification of laser-induced eyelid tightening, a force sensor (force sensor S 524060, LD Didactic GmbH, Hürth, Germany) was used in conjunction with a computer-assisted measuring system (Sensor-CASSY 2, LD Didactic GmbH, Hürth, Germany). For the evaluation of laser-induced tightening, the tension on the eyelid was measured immediately before laser irradiation, after placing the first, second, and third coagulation, as well as 5, 10 and 15 min after the third coagulation. 

### 4.3. Evaluation of the Laser-Induced Eyelid Shortening

To determine the laser-induced eyelid shortening, the macroscopically visible total longitudinal shortening of the eyelid was obtained from the difference in the length of the treated area before and after laser irradiation. For this, macroscopic photographs of the porcine eyelids were taken with a Nikon D90 camera before and 15 min after laser coagulation. A ruler was photographed with the tissue sample as a scale, and readings were taken from these images.

### 4.4. Histology

After tension measurements, porcine eyelids were fixed in 4% (*v*/*v*) paraformaldehyde (Roti Histofix^®^, Roth, Karlsruhe, Germany) for 48 h. Samples were dehydrated, embedded in paraffin, and sections of 4.5 µm thickness were cut. Hematoxylin-Eosin (HE) staining was performed to analyze the coagulation depth and diameter, as well as morphological changes of the conjunctival stroma and epithelium. Pictures of 10 slides per eyelid were taken with a light microscope (DM4000B11888804, Leica Microsystems CMS GmbH, Wetzlar, Germany), and the maximum depth and diameter was measured using ImageJ (Wayne Rasband, National Institutes of Health, Bethesda, MD, USA) (Figure 5C). The percentage of coagulated tissue relative to the total eyelid thickness and the percentage of tarsus thickness were calculated. Both thicknesses were determined in the respective slides using Image J. A grading system was established to evaluate any morphological changes and defects of the conjunctival stroma and epithelium resulting from laser irradiation (Table 2). Grade 0 was defined as no or only minor epithelial detachment (0 to 15% of the irradiated area), with no visible destruction of the underlying tissue. Minimal to moderate epithelial detachment (15 to 40% of the irradiated area), without any destruction of the underlying tissue, was classified as grade 1. Advanced epithelial detachment (40 to 70% of the irradiated area) without any visible damage to the underlying tissue, was defined as grade 2, and severe or complete epithelial detachment (70 to 100% of the irradiated area) with visible destruction of the underlying tissue was defined as grade 3.

Picro-Sirius Red Stain and a polarizing microscope (LEICA TYPE 020-519, DMLB 100S, Leica Microsystems Wetzlar GmbH, Wetzlar, Germany) were used to distinguish the irradiated areas into two differently delineated birefringent zones: a coagulated zone and a transition zone (also known as “halo”). The volume of the zones were approximated by 1/2 volume of an ellipsoid with radius *r* and height *d* with the following equation:

$$coagulated\;zone=\left(\frac{4}{3}\;\times \pi r_{2}^{2}d_{2}\right)\;÷2\;transition\;zone=\left[\left(\frac{4}{3}\;\times \pi r_{1}^{2}d_{1}\right)\right]\;÷2-coagulated\;zone$$

$r_{1}$ = radius transition zone; $r_{2}$ = radius coagulated zone

$d_{1}$ = depth transition zone; $d_{2}$ = depth coagulated zone

### 4.5. Statistics

The ImageJ software was used to collect the measurement data. Data analysis for mean and standard deviation was performed using Excel^®^ (Microsoft Corporation, Redmond, Washington, DC, USA). Statistical evaluations were carried out using GraphPad Prism 9 software (Graphpad Software Inc., San Diego, CA, USA). According to the data sets, a one-way ANOVA or two-way ANOVA with a post hoc test (Tukey, Sidak or Dunnett) was generated.

## 5. Conclusions

Laser coagulation leads to lower eyelid shortening and the increase of lower eyelid tension. The strongest effect with the least tissue damage was shown for laser parameters of 1470 nm/2.5 W/2 s. In vivo studies of the long-term effect of lasercanthoplasty have to confirm the efficacy of this concept prior to clinical application.

## Figures and Tables

**Table 1:** Treatment groups (n = 6) with the different laser parameter combinations.

Group	Wavelength [nm]	Power [W]	Duration [s]	Energy [J]
I	1940	2.5	2	5
II	1940	1	5	5
III	1470	2.5	2	5
Control	No irradiation applied			

**Table 2:** Grading system to evaluate the severity of morphologic changes of the porcine conjunctiva of the eyelid after laser irradiation. Scale bar: 500 µm.

Severity Level	Epithelial Detachment	Tissue Destruction	Representative
0	None or minor	none	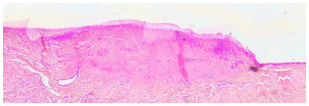
1	Minimal to moderate	none	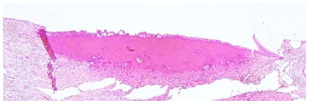
2	Advanced	none	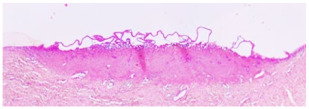
3	Maximal	existent	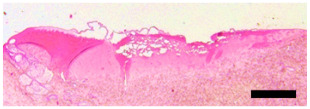

## Data Availability

Data available upon request.
